# Tanreqing Injection Attenuates Lipopolysaccharide-Induced Airway Inflammation through MAPK/NF-*κ*B Signaling Pathways in Rats Model

**DOI:** 10.1155/2016/5292346

**Published:** 2016-06-05

**Authors:** Wei Liu, Hong-li Jiang, Lin-li Cai, Min Yan, Shou-jin Dong, Bing Mao

**Affiliations:** Pneumology Group, Department of Integrated Traditional Chinese and Western Medicine, West China Hospital, Sichuan University, Chengdu 610041, China

## Abstract

*Background*. Tanreqing injection (TRQ) is a commonly used herbal patent medicine for treating inflammatory airway diseases in view of its outstanding anti-inflammatory properties. In this study, we explored the signaling pathways involved in contributions of TRQ to LPS-induced airway inflammation in rats.* Methods/Design*. Adult male Sprague Dawley (SD) rats randomly divided into different groups received intratracheal instillation of LPS and/or intraperitoneal injection of TRQ. Bronchoalveolar Lavage Fluid (BALF) and lung samples were collected at 24 h, 48 h, and 96 h after TRQ administration. Protein and mRNA levels of tumor necrosis factor- (TNF-) *α*, Interleukin- (IL-) 1*β*, IL-6, and IL-8 in BALF and lung homogenate were observed by ELISA and real-time PCR, respectively. Lung sections were stained for p38 MAPK and NF-*κ*B detection by immunohistochemistry. Phospho-p38 MAPK, phosphor-extracellular signal-regulated kinases ERK1/2, phospho-SAPK/JNK, phospho-NF-*κ*B p65, phospho-IKK*α*/*β*, and phospho-I*κ*B-*α* were measured by western blot analysis.* Results*. The results showed that TRQ significantly counteracted LPS-stimulated release of TNF-*α*, IL-1*β*, IL-6, and IL-8, attenuated cells influx in BALF, mitigated mucus hypersecretion, suppressed phosphorylation of NF-*κ*B p65, I*κ*B-*α*, ΙKK*α*/*β*, ERK1/2, JNK, and p38 MAPK, and inhibited p38 MAPK and NF-*κ*B p65 expression in rat lungs.* Conclusions*. Results of the current research indicate that TRQ possesses potent exhibitory effects in LPS-induced airway inflammation by, at least partially, suppressing the MAPKs and NF-*κ*B signaling pathways, in a general dose-dependent manner.

## 1. Introduction

Tanreqing injection (TRQ) is a widely used classical compound herbal recipe for several decades in China. It is composed of water soluble natural extractives from five crude herbal plants, namely, Radix Scutellariae Baicalensis, Fel Selenarcti, Cornu Naemorhedi, Flos Lonicerae, and Forsythiae Fructus [1], and is a mixture of about 12 main active pharmaceutical ingredients including* chlorogenic acid*,* caffeic acid*,* luteoloside*,* forsythiaside*,* forsythin*,* forsythigenol*,* baicalin*,* wogonoside*,* wogonin*,* salidroside*, and* ursodeoxycholic acid* [2]. Clinical evidence has supported the minimal toxicity and side effects of TRQ [3, 4]. With its predominant antibacterial and antiviral actions being proved by modern pharmacologic studies [5, 6], TRQ is predominately used for acute inflammatory lung diseases including acute upper respiratory tract infections [7], pneumonia [3, 8, 9], acute COPD [4, 10, 11], SARS (Serious Acute Respiration Symptom) [12, 13], A/H1N1 flu [14, 15], A/H7N9 flu [16], and the recently mentioned Middle East Respiratory Syndrome (National Health and Family Planning Commission of China, http://www.nhfpc.gov.cn/).

Stimulation of MAPKs and IKK/I*κ*B/NF-*κ*B pathways is common phenomena in many types of inflammation response. Targeting the pathways has been considered as an effective therapeutic approach to mitigate progression of numbers of inflammatory disorders, such as the classical neutrophil-predominant airway inflammation induced by Lipopolysaccharide (LPS) [17]. The involvement of the ubiquitous nuclear transcription factor NF-*κ*B in the pathogenesis of the LPS-induced inflammatory response has been well recognized [18]. LPS liberates and activates NF-*κ*B mostly through I*κ*B kinase- (IKK-) dependent phosphorylation and subsequent degradation of I*κ*B-*α* [19]. The liberated NF-*κ*B dimers are crucial for regulating transcription of diverse genes coding for cytokines including TNF-*α*, IL-1*β*, IL-6, and IL-8 [20, 21], which together contribute to the upregulation of inflammatory responses. Similar to IKK/I*κ*B-*α*/NF-*κ*B pathway, the mitogen-activated protein kinase (MAPK) pathway is also activated during an LPS-challenged inflammatory condition [22]. The three subfamilies, extracellular regulated kinases (ERK1/2), c-Jun N-terminal kinases (JNK), and p38 MAP kinases, have been implicated in the release of proinflammatory cytokines [23, 24] and the activation of NF-*κ*B [25].

Prior studies have given consistent results on the ability of TRQ to alleviate LPS-induced inflammation through multiple actions including scavenging excessive oxygen free radicals, suppressing the activation and expression of NF-*κ*B, decreasing serum NO level, and downregulating the expression of Caspase-3 and Bcl-2/Fas gene [12, 13, 26–28]. Collectively, no efforts have been done to understand the pathways that are involved in its effect. Since MAPKs and NF-*κ*B pathway has been highlighted during the LPS-induced inflammatory disorders, we hypothesize that protective actions of TRQ are likely, at least partially, due to its regulations on the two pathways. In the present study, we attempted to elucidate the anti-inflammatory potential of TRQ on LPS-induced airway inflammation in rat models by investigating the pivotal molecular basis involved in the two classical signaling pathways.

## 2. Material and Methods 

### 2.1. Animals and Drugs

75 male specific-pathogen-free (SPF) grade SD rats (10–12 weeks old, initially weighing 180–220 g) were raised under SPF conditions on a 12 h light/dark cycle at a temperature of 22 ± 2°C with free access to food and water. Protocol of this experiment was approved by the Animal Care and Use Committee of West China Hospital and the investigation conformed to the “Guide for the Care and Use of Laboratory Animals” [29]. SD rats used for the experiment were obtained from Jianyang Animal and Science Co., Ltd. (Sichuan, China). Tanreqing injection was purchased from Shanghai Kai Bao Pharmaceutical Co., Ltd. (Shanghai, China).

### 2.2. Experimental Groups and Protocol

Rats were randomly divided into four groups (*n* = 15 per group): (a) control group: rats receiving sterile saline; (b) LPS group: rats receiving intratracheal instillation (i.t.) of LPS + intraperitoneal injection (i.p.) of sterile saline; (c) low dose TRQ group: rats receiving LPS i.t. + 2.8 mL/kg TRQ i.p.; and (d) high dose TRQ group: rats receiving LPS i.t. + 5.6 mL/kg TRQ i.p. LPS (Sigma, St. Louis, MO, USA) was administered intratracheally during inspiration once at baseline, at a dose of 240 *μ*g/rat. TRQ would be injected 1 h before LPS administration at baseline and then every 24 hours.

### 2.3. Bronchoalveolar Lavage Fluid (BALF) and Tissue Extraction

On 24 h, 48 h, and 96 h, which have been previously reported as optimal time points to observe the anti-inflammatory activity of TRQ [28], rats were intraperitoneally anaesthetised with 4% sodium pentobarbital (40 mg/kg) and then exsanguinated from the abdominal aorta. The chest cavity was opened by a midline incision to expose and cannulate the trachea. The right lobe of the lung was ligated at the hilus of the lung, and the left lung was immediately lavaged three times with 2 mL ice sterile saline through the tracheostomy tube. Fluid recovery was always above 90% of the original volume. Pooled BALF samples were centrifuged and the supernatants were collected and stored at −80°C for cytokine ELISA. The deposited cells were resuspended for total leukocyte count and the differential leukocyte classification, counting 500 cells from each rat. An experienced investigator who was independent of experimental operations did all the enumerations based on standard morphological criteria. The right middle lobe of the lung was preserved in 4% paraformaldehyde for 24 h at 4°C for histology and histochemical studies. The right posterior lobe of the lung was snap preserved in liquid nitrogen and stored at −80°C for mRNA and protein analysis.

### 2.4. Enzyme-Linked Immunosorbent Assay (ELISA)

Quantitation of cytokines in the supernatants of undiluted BALF and lung homogenate was determined by ELISA technique, according to the manufacturer's instructions. Three replicates were carried out for each of the different treatments. Rat IL-1*β* ELISA kit was purchased from Shanghai ExCell Biology Company (Shanghai, China) (Cat. Number: ER008-96; Sensitivity: 15 pg/mL; Assay Range: 31.25~2000 pg/mL). Rat CXCL-1/CINC-1 ELISA kit (rat analogue of human IL-8) (Cat. Number: RCN100; Sensitivity: 1.3 pg/mL; Assay Range: 7.8~500 pg/mL), Rat IL-6 ELISA kit (Cat. Number: R6000B; Sensitivity: 0.7 pg/mL; Assay Range: 3.1~700 pg/mL), and Rat TNF-*α* ELISA kit (Cat. Number: RTA00; Sensitivity: 5 pg/mL; Assay Range: 12.5~800 pg/mL) were purchased from USA R&D Systems (Minneapolis, MN, USA). Samples were applied to wells of 96-well polystyrene microtiter plates that were precoated with specific monoclonal antibodies before incubation, and then wells were washed five times, followed by incubation with the respective HRP-conjugated polyclonal antibodies. After the repeat of aspiration and washing steps, the substrate solutions and stop solutions were added one after another. The optical density of each well was determined at 450 nm using a microplate reader (Bio-Rad, Richmond, CA) within 30 minutes.

### 2.5. Histopathology and Immunohistochemistry

Fixed specimen was rinsed in PBS, dehydrated, and embedded in paraffin according to standard procedures and serially sectioned at 4 micrometer. Then sections were stained with haematoxylin and eosin (H&E) and Alcian blue (AB)/periodic acid-Schiff (PAS) for general morphology evaluation, which were subsequently practiced by a pathologist who was blinded to group allocation under a light microscopy. The evaluation of inflammation lesions was performed using a subjective numeric scale ranging from 0 to 10, which comprises three scoring parts including peribronchial/peribronchiolar inflammation score, perivascular inflammation score, and alveolar inflammation score. Peribronchial/peribronchiolar and perivascular inflammations were individually scored from 0 to 4, representing normal (score 0), mild inflammation (score 1, <25%), moderate inflammation (score 2, 25–50%), severe inflammation (score 3, 50–75%), and very severe inflammation (score 4, >75%), respectively [30]. Alveolar inflammation was scored from 0 to 2 that represents normal (score 0), mild inflammation infiltration (score 1, few foci present), and severe inflammation infiltration (score 2, many foci present). The scores were then summed to give a total inflammatory score. Percentage of AB/PAS positively stained areas to the total area of bronchial epithelium was measured.

For p38 MAPK and NF-*κ*B immunohistochemical staining, paraffin-embedded sections were deparaffinized, rehydrated, and washed with distilled water. The monoclonal antibodies used were as follows: rabbit anti-p38 MAPK (1 : 400 dilution) and rabbit NF-*κ*B p65 (1 : 800 dilution). Both antibodies were purchased from Cell Signaling (Danvers, MA, USA). PBS was used to replace the primary antibody as a blank control. The mean optical density (IOD) was calculated by measuring 10 consecutive visual fields for each sample at a magnification of 400x, using an optical microscope equipped with an Image-Pro Plus software (version 6.0, Media Cybernetics, Silver Spring, MD, USA) by a pathologist who was blinded to the identity of the groups.

### 2.6. RNA Extraction, Reverse Transcription, and Quantitative Real-time Polymerase Chain Reaction

Frozen tissue was ground to a fine powder in liquid nitrogen. After the samples were thawed, total RNA was isolated from 30 mg lung tissue using the E.Z.N.ATM HP total RNA kit (Omega Biotech, Norcross, GA, USA) according to specific modifications to maximize RNA extraction. RNA pellets were ethanol-precipitated, washed, and resuspended in sterile ribonuclease-free water. RNA samples were reverse transcribed using the iScript cDNA synthesis kit (Bio-Rad Laboratories, Hercules, CA, USA) to synthesize the first-strand cDNA. Reverse transcription was then performed on 1 *μ*L RNA sample by adding iScript reagents to a final reaction volume of 20 *μ*L. The RNA samples were incubated in a Bio-Rad DNA Engine Peltier Thermal Cycler (Bio-Rad Laboratories, Inc., Hercules, CA, USA) at 25°C for 5 min and then reverse transcribed at 42°C for 30 min and, finally, the enzyme was denatured at 85°C for 5 min. Subsequently, the RNA concentrations were determined by measuring the absorbance at 260 and 280 nm, using a Nanodrop spectrophotometer (Montchanin, DE, USA).

PCR primers specific for selected target genes were predesigned and validated (Table 1). Gradient PCRs were used to determine the optimal annealing temperature and primer concentration. qPCR reactions had a final volume of 10 *μ*L and contained 1 *μ*L of cDNA, 0.5 *μ*L of each primer, 5 *μ*L SsoFast EvaGreen supermix (Bio-Rad Laboratories, Hercules, CA, USA), and 3 *μ*L DEPC-treated sterile distilled water. The relative expression levels of mRNA of studied cytokines were calculated relative to *β*-actin. Each qPCR was performed in triplicate for the individual sample. The PCR program was initiated by a 30 s of enzyme activation at 95°C and then 5 s of cDNA denaturation at 95°C, followed by 40 cycles at 55°C for 20 s of annealing/extension using Roche LightCycler® 96 Real-Time PCR System. A melting-point curve was then measured, starting from 65°C and increasing by 4.4°C every second up to 95°C, to detect any nonspecific PCR products. Data was analysed using 2^−ΔΔCt^ method with actin as the reference gene.

### 2.7. Western Blot Analysis

Nuclear and cytosolic extracts were prepared using a Nuclear and Cytoplasmic Protein Extraction Kit (Beyotime Co., Jiangsu, China) according to the manufacturer's instructions. The concentration of the protein was measured using a bicinchoninic acid protein assay kit (Beyotime Co., Shanghai, China). A total of 50 ug of protein that resolved with 2x SDS-PAGE was transferred onto immunoblot polyvinylidene difluoride membranes (Bio-Rad Laboratories, Inc., Hercules, CA, USA). The blots were blocked with 5% BSA in Tris-buffered saline with 0.1% Tween (TBST) for 2 h at room temperature and were incubated overnight at 4°C with primary antibodies of phospho-p38 MAPK (1 : 1000), phospho-p65 (1 : 1000), phospho-ERK1/2 (1 : 1000), phosphor-IKK*α*/*β* (1 : 900), phospho-JNK (1 : 900), phospho-I*κ*B-*α* (1 : 800), and *β*-actin (1 : 2000). Blots were then washed three times for 5 min each in TBST and incubated with horseradish peroxidase-labeled secondary goat anti-rabbit antibody at room temperature. After washing for three times, membranes were visualized with Clarity Western ECL Substrate (Bio-rad, Hercules, CA, USA). Densitometry was carried out using Quantity One (version 4.6.2) quantitation software (Bio-Rad, Hercules, CA, USA). All primary antibodies were purchased from Cell Signaling Technology (Danvers, MA, USA). *β*-actin and secondary antibody were purchased from Santa Cruz Biotechnology (Santa Cruz, CA, USA).

### 2.8. Statistical Analysis

Continuous variables were presented as mean ± standard deviation (SD) of 3 independent experiments done in duplicate. One-way analysis of variance (ANOVA) was performed to calculate the significance between groups. Pair-wise comparisons of four data sets were performed using Fisher LSD tests. The Kruskal-Wallis nonparametric ANOVA test was used for variables that do not follow a normal distribution when comparing multiple groups. A *P* value of less than 0.05 was considered statistically significant. All statistical analyses were processed using commercially available software package SPSS 20.0 (IBM SPSS Inc., Chicago, IL, USA).

## 3. Results

### 3.1. TRQ Protects LPS-Induced Histopathological Damage of Rat Lungs

**Figure 1:**
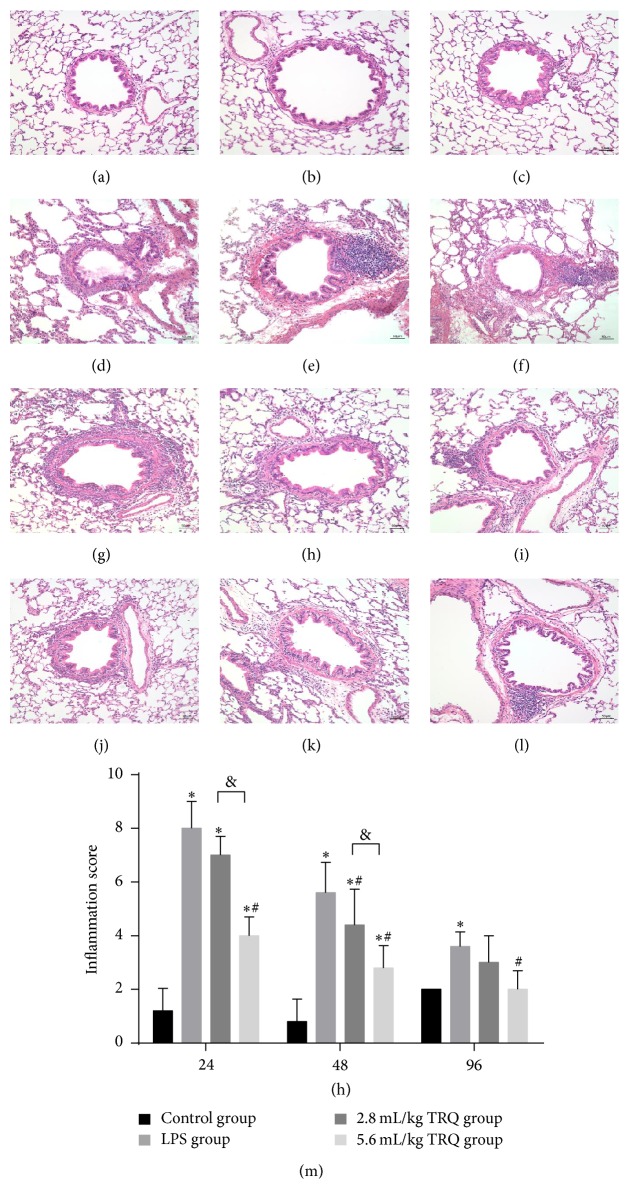
Histological changes in rat airways. Lung tissues from control rats at (a) 24 h, (b) 48 h, and (c) 96 h, rats exposed to LPS alone at (d) 24 h, (e) 48 h, and (f) 96 h, rats treated with LPS + TRQ 2.8 mL/kg at (g) 24 h, (h) 48 h, and (i) 96 h, and rats treated with LPS + TRQ 5.6 mL/kg at (j) 24 h, (k) 48 h, and (l) 96 h were all analysed by haematoxylin and eosin staining. Scale bars = 50 *μ*m. (m) Lung inflammatory scores for rat airways. Values are expressed as mean ± SD. ^*∗*^ *P* < 0.05 means significant difference from the control group; ^#^ *P* < 0.05 means significant difference from the LPS group and ^&^ *P* < 0.05 means significant difference between LPS + 2.8 mL/kg TRQ group and LPS + 5.6 mL/kg TRQ group.

Histopathological changes in rat lungs showed major difference in gross morphology between groups treated with and without TRQ. In non-LPS-exposed tissues sections, no obvious histological abnormalities were revealed (Figures 1(a)–1(c)). In contrast, intratracheal instillation of LPS induced an acute bronchopneumonia involving the focal areas of the main bronchus and also preterminal bronchioles, presented as prominent thickening of the airway epitheliums and the alveolar septa, conspicuous peribronchial inflammatory cell infiltration, and bronchiolar lumen obstruction by mucus and cell debris. In addition, some blood vessels in affected regions also thickened with a mixed inflammatory infiltrate of main neutrophils and less monocytes and lymphocytes (Figures 1(d)–1(f)). Low dose TRQ treatment failed to contribute to a remarkable alleviation of extensive inflammation with alveolar air spaces flooded with fibrinous exudate admixed with numerous neutrophils at 24 h (Figure 1(g)); however, high dose TRQ expressed noteworthy anti-inflammation property throughout the observing period (Figures 1(j)–1(l)). In general, inflammatory lesion scores decreased over time and they were effectively decreased by TRQ administration in a dose-dependent way (Figure 1(m)).

**Figure 2:**
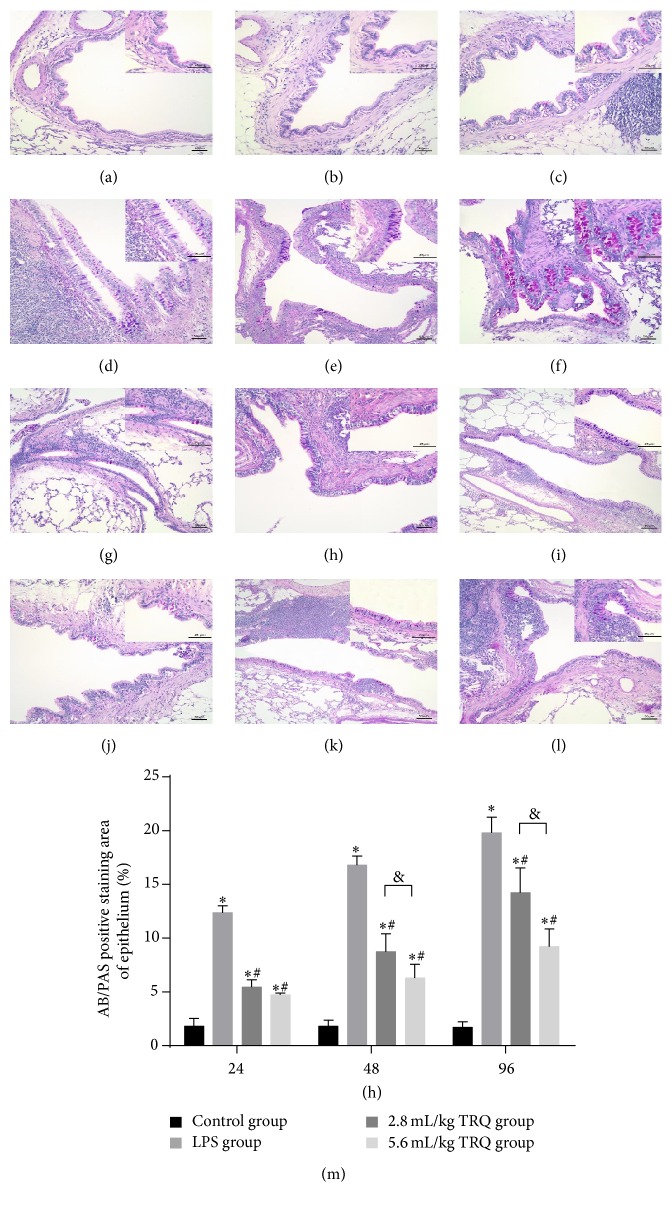
Changes of Alcian blue (AB)/periodic acid-Schiff (PAS) staining in rat airways. Lung tissues from control rats at (a) 24 h, (b) 48 h, and (c) 96 h, rats exposed to LPS alone at (d) 24 h, (e) 48 h, and (f) 96 h, rats treated with LPS + TRQ 2.8 mL/kg at (g) 24 h, (h) 48 h, and (i) 96 h, and rats treated with LPS + TRQ 5.6 mL/kg at (j) 24 h, (k) 48 h, and (l) 96 h were all analysed by AB/PAS staining. Scale bars = 50 *μ*m; upper right insert: scale bars = 25 *μ*m. (m) The percentage of AB/PAS positively staining area to total epithelial area in rat airways. Values are expressed as mean ± SD. ^*∗*^ *P* < 0.05 means significant difference from the control group; ^#^ *P* < 0.05 means significant difference from the LPS group and ^&^ *P* < 0.05 means significant difference between LPS + 2.8 mL/kg TRQ group and LPS + 5.6 mL/kg TRQ group.

Besides, a noticeable and conspicuous increase in numbers of mucous cells and amounts of mucosubstances was detected along the airway surface epithelium in LPS group (Figures 2(d)–2(f)), verifying the exact link between airway inflammation and mucus production [31], while the goblet cell metaplasia and hyperplasia were rarely found in the main airways in control rats (Figures 2(a)–2(c)), with a faint positive AB/PAS staining area being detected. TRQ treatment significantly inhibited, though not fully abrogated, the mucus hypersecretion at each time point (Figures 2(g)–2(l)).

### 3.2. TRQ Regulates LPS-Induced Leukocyte Accumulation in BALF

**Figure 3:**
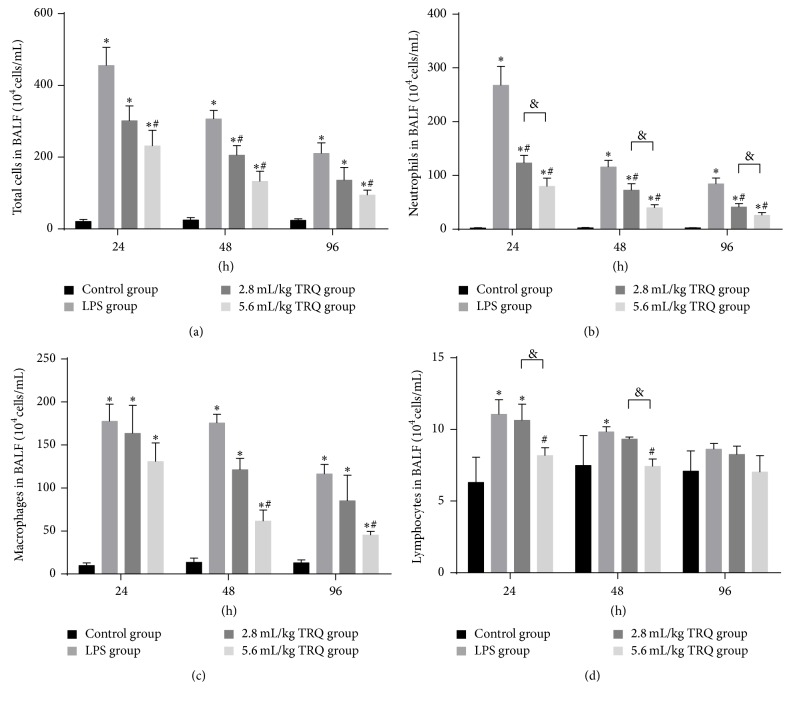
Total and differential cell counts in BALF. ^*∗*^ *P* < 0.05 means significant difference from the control group; ^#^ *P* < 0.05 means significant difference from the LPS group and ^&^ *P* < 0.05 means significant difference between LPS + 2.8 mL/kg TRQ group and LPS + 5.6 mL/kg TRQ group.

As a potent stimulus for immune cell, LPS significantly recruited more cells at 24 h, and although the total cell amount decreased over time, it was always significantly higher than that in the control group. TRQ effectively accelerated the process of cell number decrease (Figure 3(a)). For the number of neutrophils, even low dose of TRQ effectively cut down the neutrophil release in BALF after LPS administration at 24 h (Figure 3(b)). The inhibitory effect of TRQ in macrophage accumulation started from 48 h, which was only in high dose group (Figure 3(c)). The number of lymphocytes in BALF enhanced observably after LPS administration, which was completely reversed by high dose TRQ at 24 h (Figure 3(d)).

### 3.3. TRQ Attenuates LPS-Induced Proinflammatory Cytokines in BALF and Lungs

**Figure 4:**
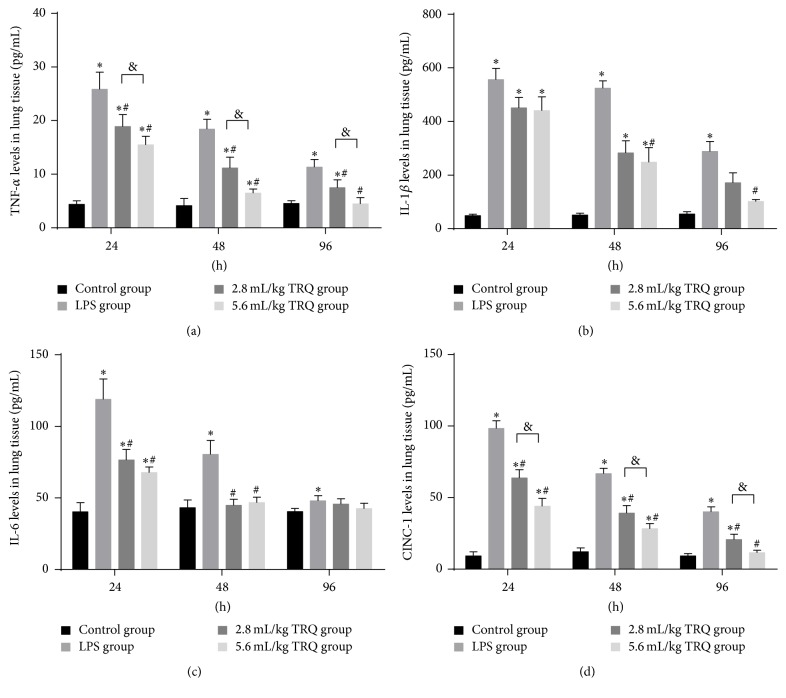
Levels of cytokines in lung tissues. ^*∗*^ *P* < 0.05 means significant difference from the control group; ^#^ *P* < 0.05 means significant difference from the LPS group and ^&^ *P* < 0.05 means significant difference between LPS + 2.8 mL/kg TRQ group and LPS + 5.6 mL/kg TRQ group.

**Figure 5:**
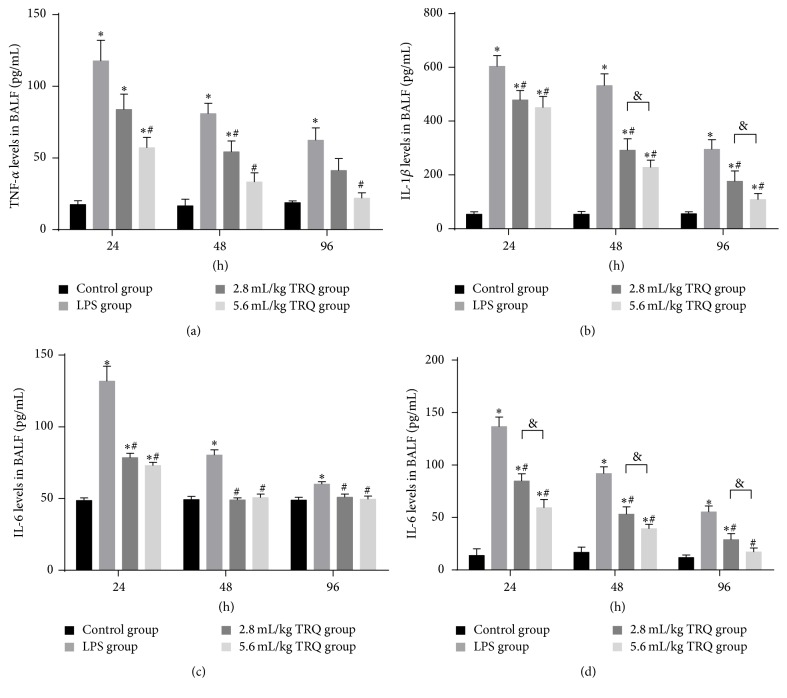
Levels of cytokines in BALF. ^*∗*^ *P* < 0.05 means significant difference from the control group; ^#^ *P* < 0.05 means significant difference from the LPS group and ^&^ *P* < 0.05 means significant difference between LPS + 2.8 mL/kg TRQ group and LPS + 5.6 mL/kg TRQ group.

Since they have been previously reported [32], protein release and mRNA upregulation of TNF-*α*, IL-1*β*, IL-6, and CINC-1 were examined to determine the effect of TRQ. As expected, levels of selected cytokines were significantly elevated in BALF (Figure 4) and lung homogenate (Figure 5) 24 h after LPS inoculation. Although they were always significantly higher than those in the control groups, levels of TNF-*α*, IL-6, and CINC-1 tended to rapidly decrease from 24 h, while levels of IL-1*β* continued to be high at 48 h. Amounts of TNF-*α*, IL-6, and CINC-1 were markedly reduced starting from 24 h after LPS exposure by even low dose of TRQ. Of interest, for IL-1*β* a reduction was observed only in high dose TRQ group at 48 h. At the last time point, high dosage of TRQ successfully recovered levels of all cytokines to normal. Taken together, TRQ exerted a more potent and earlier effect on reversing the overproduction of IL-6, which was completely repressed at 48 h.

**Figure 6:**
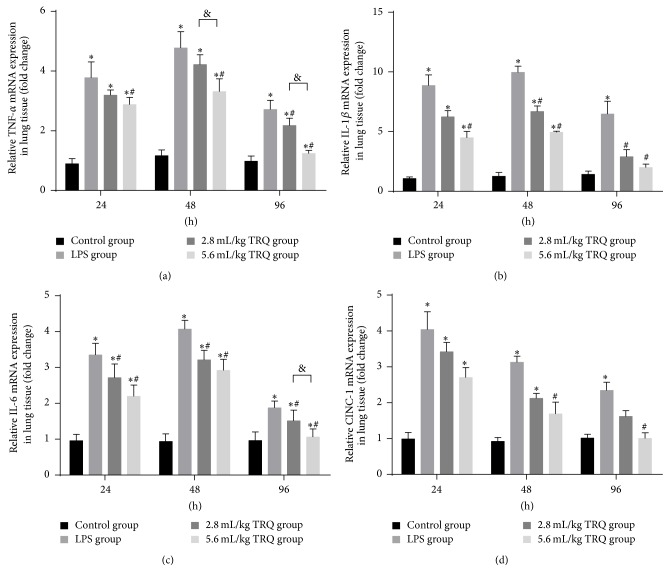
Changes in relative mRNA levels of cytokines in lung tissues. ^*∗*^ *P* < 0.05 means significant difference from the control group; ^#^ *P* < 0.05 means significant difference from the LPS group and ^&^ *P* < 0.05 means significant difference between LPS + 2.8 mL/kg TRQ group and LPS + 5.6 mL/kg TRQ group.

As for the qPCR results, CINC-1 was accumulated with mRNA levels peaking at 24 h and then declining. TNF-*α*, IL-6, and IL-1*β* mRNA reached a plateau at 24 h and continued to escalate until the end of the 48 hour study. These data demonstrated the complex patterns of cytokine gene expression and suggest that production of early mediators may augment continued expression of TNF-*α*, IL-6, and IL-1*β* mRNA. The mRNA induction for each cytokine was significantly mitigated or reversed by TRQ treatment (Figure 6).

### 3.4. TRQ Modulates LPS-Induced Synthesis of Signaling Proteins in Rat Lungs

**Figure 7:**
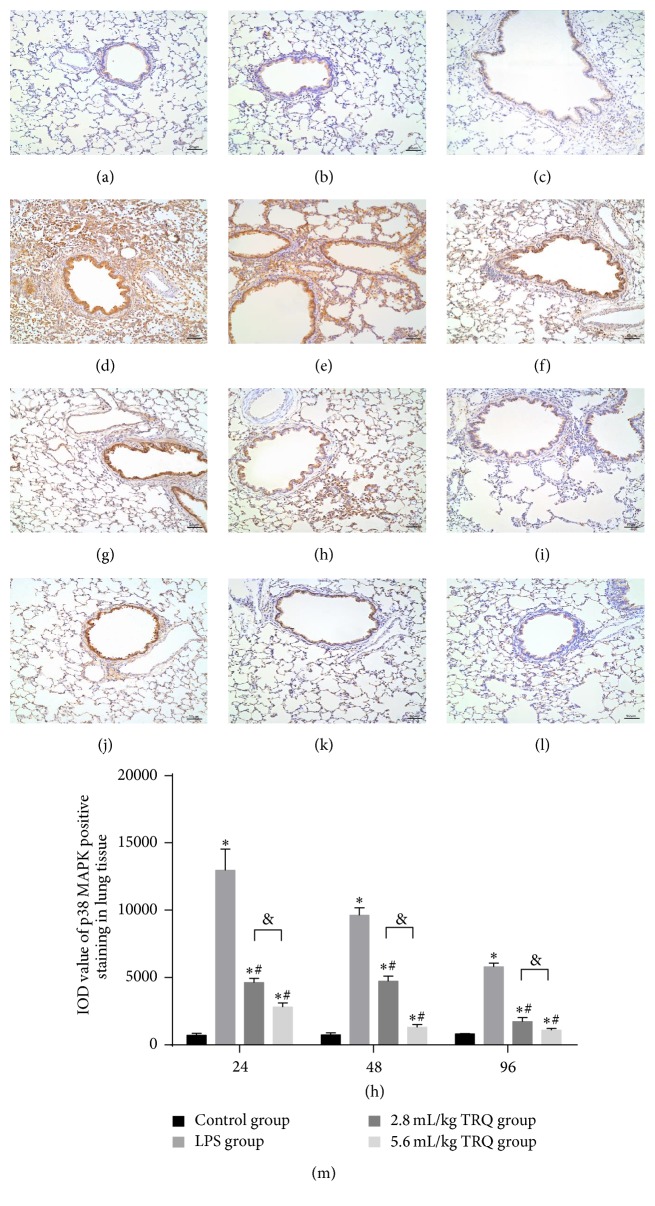
Changes in p38 MAPK immunohistochemical staining in rat airways. Lung tissues from control rats at (a) 24 h, (b) 48 h, and (c) 96 h, rats exposed to LPS alone at (d) 24 h, (e) 48 h, and (f) 96 h, rats treated with LPS + TRQ 2.8 mL/kg at (g) 24 h, (h) 48 h, and (i) 96 h, and rats treated with LPS + TRQ 5.6 mL/kg at (j) 24 h, (k) 48 h, and (l) 96 h were all analysed by haematoxylin and eosin staining. Scale bars 50 *μ*m. (m) Integrated option density sum (IOD sum) value of positive p38 MAPK staining in rats. Values are expressed as mean ± SD. ^*∗*^ *P* < 0.05 means significant difference from the control group; ^#^ *P* < 0.05 means significant difference from the LPS group and ^&^ *P* < 0.05 means significant difference between LPS + 2.8 mL/kg TRQ group and LPS + 5.6 mL/kg TRQ group.

**Figure 8:**
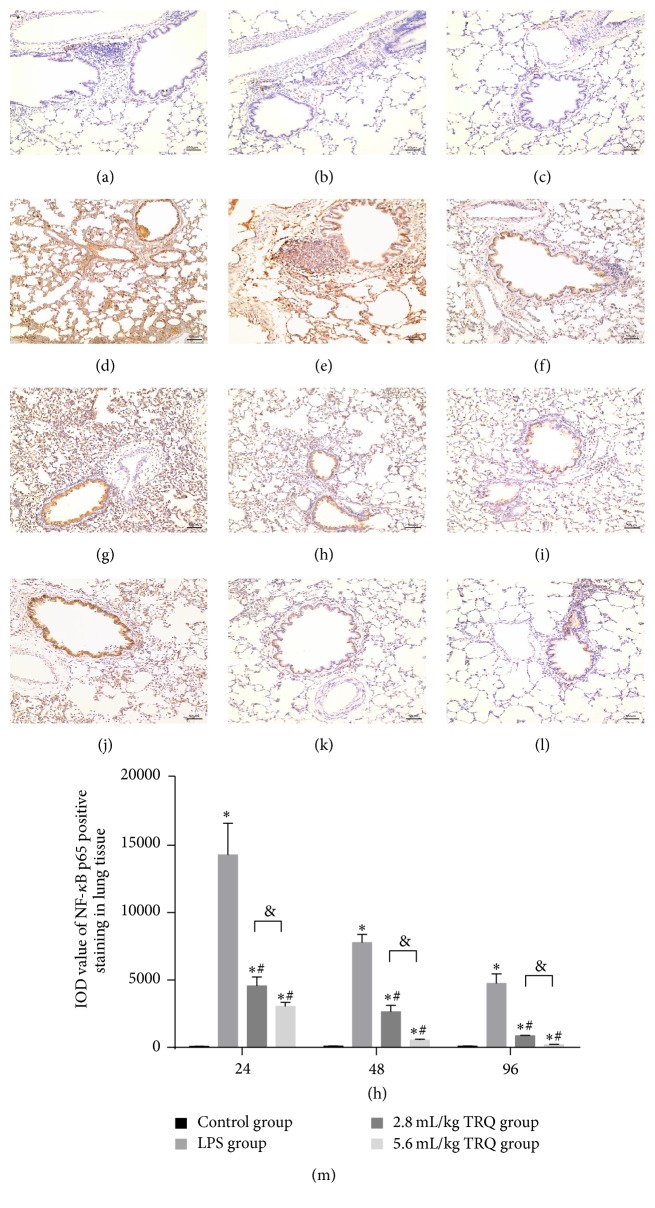
Changes in NF-*κ*B p65 immunohistochemical staining in rat airways. Lung tissues from control rats at (a) 24 h, (b) 48 h, and (c) 96 h, rats exposed to LPS alone at (d) 24 h, (e) 48 h, and (f) 96 h, rats treated with LPS + TRQ 2.8 mL/kg at (g) 24 h, (h) 48 h, and (i) 96 h, and rats treated with LPS + TRQ 5.6 mL/kg at (j) 24 h, (k) 48 h, and (l) 96 h were all analysed by haematoxylin and eosin staining. Scale bars 50 *μ*m. (m) Integrated option density sum (IOD sum) value of positive NF-*κ*B p65 staining in rats. Values are expressed as mean ± SD. ^*∗*^ *P* < 0.05 means significant difference from the control group; ^#^ *P* < 0.05 means significant difference from the LPS group and ^&^ *P* < 0.05 means significant difference between LPS + 2.8 mL/kg TRQ group and LPS + 5.6 mL/kg TRQ group.

Effect of TRQ on LPS-induced p38 MAPK and NF-*κ*B p65 expression in rat lungs was measured by immunohistochemistry. Results revealed that IOD value of areas positively stained by p38 MAPK and NF-*κ*B p65 monoclonal antibody in* * lung sections increased markedly at 24 h after LPS exposure, which could be significantly attenuated by TRQ in a dose-dependent way (Figures 7 and 8).

### 3.5. TRQ Modulates LPS-Induced Phosphorylation of Signaling Proteins in MAPKs Pathway in Rat Lungs

**Figure 9:**
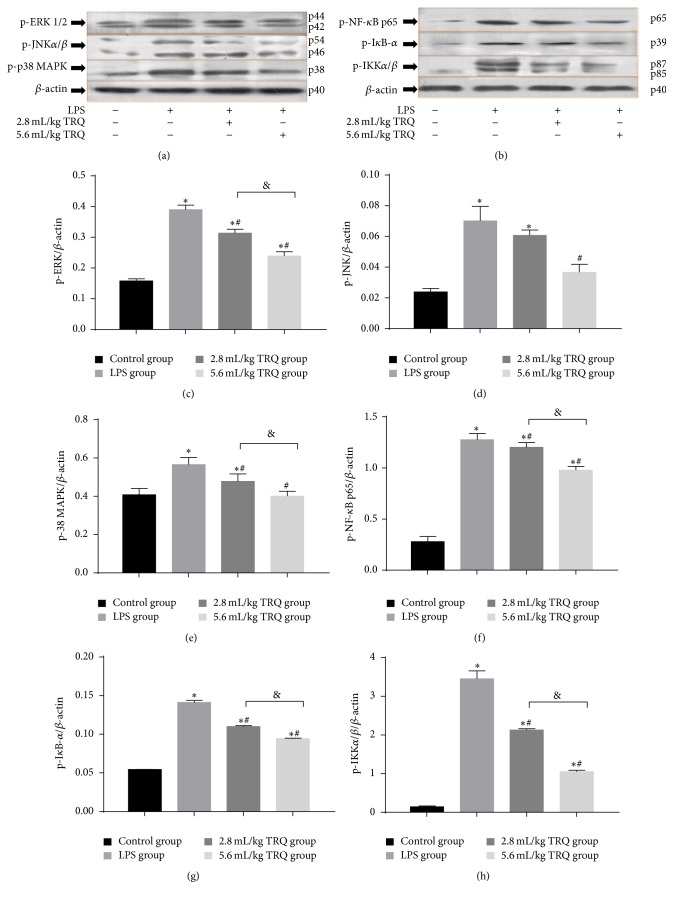
Changes in protein levels in lung tissues. ^*∗*^ *P* < 0.05 means significant difference from the control group; ^#^ *P* < 0.05 means significant difference from the LPS group and ^&^ *P* < 0.05 means significant difference between LPS + 2.8 mL/kg TRQ group and LPS + 5.6 mL/kg TRQ group.

Western blot analysis on changes of prominent protein expressions involved in MAPKs pathway in rat lungs showed that stimulation with LPS resulted in a significant increase in the amount of phosphorylation of p38, JNK, and ERK1/2 compared with the control group at 24 h, which were markedly inhibited by an addition of TRQ (Figures 9(a) and 9(c)–9(e)). Except for phospho-JNK, a significant difference between the effects in different dosage of TRQ was observed in all indicators.

### 3.6. TRQ Suppresses LPS-Induced Phosphorylation of Signaling Proteins in NF-*κ*B Pathway in Rat Lungs

According to the results, stimulation with LPS alone for 24 h notably induced the strong signal of the immunostained band for phosphorylated p65, I*κ*B-*α*, and IKK*α*/*β* in rat lungs, which were significantly decreased after TRQ treatment in a dosage-dependent manner, as expected (Figures 9(b) and 9(f)–9(h)).

## 4. Discussion

In recent decades, MAPKs and IKK/I*κ*B-*α*/NF-*κ*B p65 signaling pathways have attracted considerable attention as targets for inflammation inhibition. In the present study, we focused on the two pathways to tentatively explore the pharmacological mechanisms of TRQ, which, we hope, might in turn advance the drug development. Here we showed that TRQ effectively reduced the phosphorylation of pivotal factors in MAPKs and NF-*κ*B signaling pathways, which contribute to significantly less recruitment and infiltration of immune cells in BALF and subsequent suppression on toxic cytokines release, realizing a promising mitigation on LPS-induced airway inflammation.

As prominent target cells of LPS, neutrophils and macrophages were dramatically induced in BALF after LPS stimulation. As for the neutrophil migration, LPS induces an enhanced chemokinesis by its direct effect on cells and an increased chemotaxis by indirect effects. For example, LPS challenge prominently enhances the level of IL-8, an essential CXC chemokine to attract neutrophils to sites of inflammation in the lung. Both direct and indirect effects are modulated by the coordinated action of ERK, p38 MAPK, and NF-*κ*B pathways [33]. The pathways also contribute to macrophage migration. It has been demonstrated that LPS or TNF-*α*-induced activation of matrix metalloproteinases, key players in macrophage migration and invasion into foci inflammation, is controlled via ERK1/2, JNK, and IKK/NF-*κ*B pathway [34, 35]. Besides, the expression of MCP-1, another important chemokine for monocytes/macrophages, is stimulated by LPS via NF-*κ*B-dependent mechanism [36]. In particular, the involvement of lymphocytes was also observed after LPS treatment in this study. Previous data showed that lymphocytes present in lung played a role in immunopathology of inflammatory process in both humans and mice [37, 38]. More specifically, study of Dushianthan and colleagues [39] revealed a rapid infiltration of different T cells, including regulatory T cells (Tregs), NKT, and NK cells in human lungs in response to LPS, accompanied by a significant elevation of IL-17, a potent recruiter of neutrophils to inflammatory sites. They demonstrated that both lymphocyte released IL-17 and Tregs modulated the recruitment of neutrophils to the lung in LPS-induced ALI. Therefore, the drastic rise of neutrophils in BALF may partly be attributed to the elevation of lymphocytes in this study.

It has been well characterized that LPS administration on rats leads to overproduction of cytokines from neutrophils and macrophages including IL-1*β*, IL-6, IL-8, and TNF-*α* [40–42] for up to 48 h [43], as a result of a series of MAPKs/NF-*κ*B signal transduction cascade. It has been demonstrated that pretreatment with TNF inhibitors led to a reduction in circulating IL-1, IL-6, and IL-8, suggesting an important role of TNF-*α* in the amplification of inflammatory response [44]. TNF helps neutrophils, monocytes, and lymphocytes recruitment and infiltration by inducing vasodilation and loss of vascular permeability [45] and triggering the secretion of chemokines [46] and cell adhesion molecules [47]. Similarly to TNF, IL-1*β* is also mainly produced and released from monocytes/macrophages in the initiation of inflammatory process after endotoxin exposure. Released IL-1*β* subsequently triggers further production of IL-1*β* and other cytokines/chemokines by infiltrating cells and accounts for the perpetuation of inflammation. This may partially explain why IL-1*β* keeps at high levels at 48 h after LPS instillation in our results. As a pleiotropic cytokine that possesses both pro- and anti-inflammation properties, IL-6 plays a key role in acute-phase of lung injury by affecting the release and functions of neutrophils and macrophages [48]. Time-course studies showed that induction of mRNA for IL-1*β* and TNF-*α* occurred rapidly preceding that of IL-6 mRNA after LPS exposure [49]. Subsequently, depending on p38 MAPK and NF-*κ*B pathways, early produced IL-1*β* and TNF-*α* induced IL-6 mRNA expression and more IL-6 production. This may explain the continued rise in IL-6 mRNA level till 48 h. Therefore, it is noteworthy that a large proportion of LPS-stimulated IL-6 is actually indirectly induced by TNF-*α* and/or IL-1*β* [50]. Inversely, endotoxin-induced IL-6 functions to downregulate its own inducers, TNF-*α* and IL-1*β* on mRNA and protein levels [51, 52], with a negative feedback mechanism. As a result, the acute neutrophil and macrophage exudation would be diminished [53], realizing the protective role of IL-6 on endotoxin-induced inflammation [54]. Although nonnegligible amount of IL-6 is induced by TNF-*α* and IL-1*β* in this condition, TNF-*α* and IL-1*β* are both produced in a very rapid burst in response to LPS; therefore, the decrease of IL-6 levels here was likely not due to a secondary effect exerted by decline of TNF-*α* and IL-1*β* levels, which should be ascribed to the effect from TRQ. Clinical trials demonstrated that TRQ administration showed a potent effect on lowering the levels of IL-1*β*, IL-6, IL-8, TNF-*α*, and IL-17 in plasma of patients under acute pulmonary conditions [11, 55, 56], which was promisingly consistent with our results obtained from rat models.

To our knowledge, it is the first attempt to discover the underlying mechanism involving signaling pathways for the effect of TRQ. This present study had some limitations. Although pathogenesis of LPS-induced airway inflammation generally involves multiple signaling pathways, we only investigated MAPKs/NF-*κ*B pathways and only discussed its role in LPS-stimulated inflammations in vivo. If necessary, we will conduct more comprehensive work in the future to systematically evaluate its function in vitro and multiple pathways and also other pathogen-induced inflammation models.

## 5. Conclusion

In summary, this work demonstrated that TRQ dose-dependently attenuated LPS-induced neutrophils, macrophages, and lymphocytes infiltration and inhibited proinflammatory cytokines including TNF-*α*, IL-1*β*, IL-6, and IL-8 on both mRNA transcription and protein synthesis levels, which were very beneficial to the resolution of inflammation. The therapeutic action of TRQ, in some degree, benefited from its inhibitory effect on the expressions of p38 MAPK and NF-*κ*B p65 in lungs by reducing phosphorylation of p38 MAPK, ERK1/2, IKK*α*/*β*, I*κ*B-*α*, and NF-*κ*B p65. All the present findings specified that the underlying mechanisms of the suppressive actions of TRQ on LPS-induced airway inflammation and mucus overproduction might be due to, at least in part, the suppression on MAPKs and NF-*κ*B signaling pathways.

## Figures and Tables

**Table 1:** The primer sequences.

TNF-*α*-forward	TGCTATCTCATACCAGGAGA
TNF-*α*-reverse	GACTCCGCAAAGTCTAAGTA
IL-6-forward	TCTTGGGACTGATGTTGTTG
IL-6-reverse	TAAGCCTCCGACTTGTGAA
IL-1*β*-forward	GCAACTGTTCCTGAACTCAACT
IL-1*β*-reverse	ATCTTTTGGGGTCCGTCAACT
CXCL-1/CINC-1-forward	CTCCAGCCACACTCCAACAGA
CXCL-1/CINC-1-reverse	CACCCTAACACAAAACACGAT
*β*-actin-forward	CCT CAT GAA GAT CCT GAC CG
*β*-actin-reverse	ACC GCT CAT TGC CGA TAG TG
